# A retrospective cohort study to investigate fatigue, psychological or cognitive impairment after TIA: protocol paper

**DOI:** 10.1136/bmjopen-2015-008149

**Published:** 2015-05-02

**Authors:** Grace M Moran, Melanie Calvert, Max G Feltham, Ronan Ryan, Tom Marshall

**Affiliations:** Primary Care Clinical Sciences, University of Birmingham, Birmingham, UK

**Keywords:** transient ischaemic attack, fatigue, anxiety, depression, cognitive impairment

## Abstract

**Introduction:**

Transient ischaemic attack (TIA) is defined by short-lasting, stroke-like symptoms, and is recognised as a medical emergency. Symptoms are assumed to completely resolve, and treatment is focused on secondary stroke/TIA prevention. However, evidence suggests that patients with TIA may experience ongoing residual impairments, which they do not receive therapy for as standard practice. TIA-induced sequelae could impact on patients’ quality of life and ability to return to work or social activities. We aim to investigate whether TIA is associated with subsequent consultation for fatigue, psychological or cognitive impairment in primary care.

**Methods and analysis:**

A retrospective open cohort study of patients with first-ever TIA and matched controls. Relevant data will be extracted from The Health Improvement Network (THIN) database, an anonymised primary care database which includes data for over 12 million patients and covers approximately 6% of the UK population. Outcomes will be the first consultation for fatigue, anxiety, depression, post-traumatic stress disorder or cognitive impairment. Principal analysis will use Kaplan-Meier survivor functions to estimate time to first consultation, with log-rank tests to compare TIA and control patients. Cox proportional hazard models will predict the effect of demographic and patient characteristics on time to first consultation.

**Ethics and dissemination:**

Approval was granted by a THIN Scientific Review Committee (ref: 14-008). The study's findings will be published in a peer-reviewed journal and disseminated at national and international conferences and through social media.

## Introduction

Transient ischaemic attack (TIA) is defined by short-lasting, stroke-like symptoms which usually resolve within 1–2 h without causing cerebral infarction.^1^ TIA is associated with an increased risk of subsequent stroke, and treatment is focused on secondary stroke/TIA prevention.^2^ It is currently assumed that patients do not experience any TIA-induced sequelae; however, patients have anecdotally reported ongoing residual impairments post-TIA.^3^ Fatigue, psychological and cognitive impairments occur post-stroke and could be potential sequelae of TIA. These impairments are associated with reduced quality of life, impaired functioning and increased mortality post-stroke.^4–7^ It is important to establish the holistic consequences of TIA; if patients experience ongoing impairments, they could impact on patients’ quality of life and ability to return to work or social activities. Therefore, preventative medical management alone, without addressing residual impairments, is unlikely to be adequate. Additionally, these impairments may be subtle and missed by clinicians, but are meaningful for the patient.

We recently conducted a systematic review investigating the prevalence of fatigue, psychological and cognitive impairment following TIA and minor stroke. There was evidence to suggest these patients experience residual impairments; however, existing studies had important limitations.^8^ We were unable to determine if the prevalence of impairments post-TIA was greater than that of the general population because few studies included a control group. The association between TIA and subsequent impairments was unclear as most studies did not measure or control for presence of impairments prior to TIA or minor stroke.

This study will address the limitations of existing studies and explore if TIA is associated with subsequent fatigue, psychological or cognitive impairment. If present, there is the potential for TIA-induced impairment to increase stroke risk through biological mechanisms (such as increased blood pressure from anxiety) or behavioural change (such as non-adherence to stroke prevention medication if these drugs were attributed to post-TIA impairments). This study aims to investigate (1) whether TIA is associated with subsequent consultation for fatigue, psychological or cognitive impairment in primary care and (2) if patients with TIA who consult with these residual impairments are more likely to experience a subsequent stroke.

## Methods and analysis

### Study design

A retrospective open cohort study of patients with first-ever TIA and controls matched by age (±2 years), sex and general practice.

### Data source

Data will comprise of anonymised UK primary care patient records extracted from The Health Improvement Network (THIN). Over 500 general practices contribute to the THIN database which covers approximately 6% of the UK population and has data for over 12 million patients, including 3.6 million current patients.^9^ Practices that contribute data to THIN use Vision patient records software which codes clinical data using the Read code clinical classification (V.2)^10^ and drug prescriptions which link to the British National Formulary.^11^

### Population

**Figure 1:**
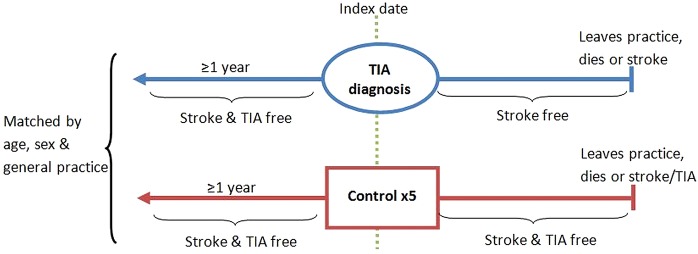
Summary of matching and eligibility criteria for transient ischaemic attack (TIA) and control patients.

Relevant data will be extracted for patients with first-ever TIA aged 18 years and over with no previous history of stroke. For each patient with TIA, we will select five^12^ controls free from stroke and TIA and matched on age (±2 years), sex and general practice. The date of TIA will be taken as the index date, and controls will be part of the same general practice as their matched patients with TIA on the index date (figure 1). Controls will be selected from the pool of potential controls without replacement to ensure they only act as a control once. Control patients who experience a TIA in follow-up will become part of the TIA group if they meet the eligibility criteria. For data quality reasons, the index date must occur between 1 January 2000 and the practice's most recent data collection, and have occurred after the practice date of acceptable mortality recording.^13^ TIA and control patients must have been registered at their practice for at least 1 year prior to diagnosis to obtain baseline data. Patients will be followed up until they leave the practice, die or suffer a TIA (control patients only) or stroke.

### Study variables

#### Outcome variables

The principal outcomes will be the first consultation for fatigue, anxiety, depression, post-traumatic stress disorder (PTSD) or cognitive impairment. The outcomes will be defined by relevant clinical codes (Read codes) for symptoms and diagnoses, or drug codes (see online supplementary appendix 1). Cognitive impairment will include memory, attention and executive functioning impairments but not dementia. The outcomes will be grouped into three categories: (1) fatigue, (2) cognitive impairment and (3) psychological impairment (comprised of anxiety, depression and PTSD). Stroke will be a secondary outcome, and the first occurrence of a stroke is a censoring event for the principal outcomes.

#### Exposures variables

A comprehensive list of clinical codes for stroke and TIA has been developed which will identify the eligible population (see online supplementary appendix 2). TIA and control patients must have no clinical codes indicating a previous stroke or TIA prior to the index date.

#### Follow-up

Follow-up of TIA and control patients will continue until the first occurrence of: death, stroke, patient leaves their practice or the last data collection from the general practice. Diagnosis of another TIA during the follow-up period will be permitted for patients with TIA; however, control patients will be censored on the date a TIA is recorded, and subsequently will become part of the TIA group. Three substudies will be formed for each outcome category (fatigue, psychological or cognitive impairment) and patients will be censored at the first consultation for the relevant outcome.

#### Predictor variables

The most recent baseline demographic and patient characteristics prior to index date will be extracted including age (at index date), sex, body mass index (BMI), Townsend deprivation quintiles,^14^ urban/rural residence,^14^ smoking status and alcohol consumption. Existing comorbidities may be associated with fatigue, psychological or cognitive impairment; therefore, comorbidities will be measured and comprise of the long-term conditions included in the Quality and Outcomes Framework (QOF), identified by their corresponding Read codes (QOF business rules V.27; see online supplementary appendix 3).^15^ Although other conditions may be potential confounders, the QOF incentives scheme means that these conditions are likely to be well recorded, and they include the majority of important conditions. Number of consultations will be reported because patients who consult more would have increased opportunity to report residual impairments. Furthermore, consultations for fatigue, psychological or cognitive impairment prior to the index date will be extracted to control for presence of the outcomes prior to the index date.

### Quality checks, missing data and extreme values

Data are unlikely to be missing at random;^16^ therefore, no attempt will be made to impute numeric missing data, and continuous variables will be categorised with an additional ‘missing’ category included. Absence of clinical codes for diagnoses will be taken to indicate the diagnosis is not present. Clinically implausible values for height, weight and BMI will be excluded with Health Survey for England statistics used as a guide.^17^

### Analysis

Data management and analysis will be performed using STATA V.12 (StataCorp, College Station, Texas, USA). The principal analysis will use Kaplan-Meier (K-M) survivor functions to estimate time to each outcome for TIA and control patients (ie, first consultation where there is a clinical code indicating fatigue, anxiety, depression, PTSD or cognitive impairment). Log-rank tests will compare TIA and control patients’ K-M survivor functions. Cox proportional hazard models will be used to predict the effect of demographic and patient characteristics on time to each outcome. Backwards elimination, with a p-to-eliminate value of >0.05, will select covariates included in the models. General practice will be included as a random effect, and age and sex will be forced into the model to adjust for residual confounding. Fatigue and cognitive impairment will be analysed individually. Anxiety, depression and PTSD will be combined as psychological impairments, but analysed individually in an exploratory analysis. Sensitivity analysis will restrict the analysis to patients with no record of fatigue, psychological or cognitive impairment prior to the index date. To investigate if patients with TIA who consult for residual impairments are more likely to have a stroke, secondary analysis will use K-M survivor functions to estimate time to first stroke for patients with TIA with and without residual impairments. An exploratory analysis will investigate the incidence of stroke in the first year post-TIA. Similar to the principal analysis, demographic and patient characteristics will be adjusted for using Cox proportional hazard models. Exploratory analysis will also investigate the effect of excluding patients with no consultations in follow-up, or those who consult for outcomes within the first month of follow-up.

## Discussion

Follow-up for patients with TIA is conducted in primary care; therefore, it is important for primary care clinicians to understand if patients experience TIA-related impairments which require additional treatment to secondary stroke prevention. A systematic review of the literature found evidence to suggest fatigue, psychological and cognitive impairment following TIA. However, the evidence was limited and the review highlighted the need for further research comprised of a large, matched cohort study.^8^ Our study will provide a valuable contribution to the literature, increase the understanding of the needs of this patient group, and potentially inform an intervention study.

This study is likely to have a large sample size, and data will be representative of ‘real-life’ primary care practice as data are collected in routine clinical care. Contrary to most existing studies in this field, we will include a matched control group and will control for the presence of fatigue, psychological and cognitive impairment prior to TIA. Limitations of the study include the accuracy of diagnosis and recording of TIA and our outcomes (fatigue, cognitive and psychological impairment) in primary care. General practitioners are incentivised to keep a register of patients with TIA;^15^ however, it has been recognised that TIA can be misdiagnosed.^18^ Although TIA may be underdiagnosed, our data will be representative of the current state of TIA diagnoses in primary care. Ideally, we would have included patients with minor stroke in our sample; however, severity of stroke is not coded in the Read clinical coding.

Our outcomes are likely to be under-reported because, although residual impairments could impact on patients’ quality of life, they may be subtle and, consequently, patients may not consult in primary care for them. Furthermore, impairments may not be recognised by primary care clinicians, for example, evidence suggests poor recognition and recording of mild cognitive impairment in primary care.^19^ However, we have developed an extensive list of clinical codes which encompass symptoms as well as diagnoses and, where possible, included related medication to define outcomes. Diagnosis of depression is incentivised by QOF and is, therefore, likely to be well recorded. General practices are expected to differ in their recording of our outcomes, and to control for this, we will match TIA and control patients on this variable. It is important to note that the THIN database comprises of primary care data; therefore, this study will include primary care consultations for fatigue, psychological and cognitive impairments rather than the incidence of these impairments in the community.

A limitation of using electronic medical records is that duration of our outcomes cannot be determined as we are unable to identify if or when symptoms resolve. Patients may experience fatigue, psychological or cognitive impairment before their index date, and have a clinical code to indicate this. However, if the impairment is still present after the index date, the presence of the impairment may not be recorded again and we will not be able to include the continued presence of this impairment in our analysis. Furthermore, patients with TIA may potentially consult more in primary care because of TIA-related follow-up appointments. This could introduce an ascertainment bias as patients with TIA would have more opportunity to report fatigue, psychological or cognitive impairments compared with those who consult less frequently. We will descriptively report the average number of consultations for TIA and control patients, and discuss the potential impact on our results.

### Dissemination

The findings of the study will be published in a peer-reviewed journal, and disseminated at national and international conferences and through social media.
